# Peritoneal metastatic adenocarcinoma possibly due to a gastric duplication cyst: a case report and literature review

**DOI:** 10.1186/1471-230X-14-48

**Published:** 2014-03-19

**Authors:** Kuiliang Liu, Xiangchun Lin, Jing Wu, Hong Liu, Mingming Meng, Hui Su, Weiping Tai, Hong Chang

**Affiliations:** 1 Gastroenterology Department, Beijing Shijitan Hospital, Capital Medical University, No.10 Tieyi road, Haidian, Beijing 100038, People’s Republic of China; 2 Pathology Department, Beijing Shijitan Hospital, Capital Medical University, No.10 Tieyi road, Beijing, Haidian 100038, People’s Republic of China

## Abstract

**Background:**

Gastric duplication cysts are rare congenital abnormalities, and malignant transformation of these duplications is also thought to be rare.

**Case presentation:**

During a routine health checkup, a 28-year-old man underwent abdominal sonography followed by computed tomography (CT) with contrast agent, which revealed a cystic lesion with no enhancement. Laparoscopic surgery showed a 10 × 10 cm cyst adhering to the gastric corpus. However, attempts to remove the lesion en bloc were unsuccessful, and the ruptured cyst had contaminated the peritoneal cavity. Gastric duplication was diagnosed from microscopic examination of the cyst. Seven months later, the patient suffered a progressive increase in ascites, and repeated cytological analysis showed small nests of adenocarcinoma cells, with primary lesion unknown. Diagnostic laparoscopy showed multiple white nodules scattered over the surface of the liver, greater omentum, and peritoneum. Biopsy of the omental nodules confirmed adenocarcinoma, while carcinomatosis was diagnosed in the peritoneum.

**Conclusions:**

Clinical presentation and chronological developments indicated that the malignancy probably originated from the gastric duplication cyst. This case highlights the importance of accurate preoperative diagnosis and optimal surgical management for gastric duplication as well as considering the potential existence of malignant transformation during surgical evaluation of adult patients with gastric duplication cysts.

## Background

Most cases of gastrointestinal duplication cysts, which are rare congenital anomalies, are identified at a very young age, and these cysts are rarely diagnosed in asymptomatic adults [1]. Malignant transformation of gastric duplications is considered rare and is seen only in the case of adult patients. Here, we describe a case of peritoneal carcinomatosis with histologic features compatible with primary upper gastrointestinal lesion, which probably originated from an incompletely removed gastric duplication cyst. In retrospect, a malignant tumor could have been present at diagnosis of the cyst 8 months earlier. Based on the present case study and the study by Zheng et al. [2], we recommend increasing the awareness about the malignant potential of gastrointestinal duplication cysts in adult patients, and we suggest that these cysts be treated as malignant tumors.

## Case presentation

**Figure 1:**
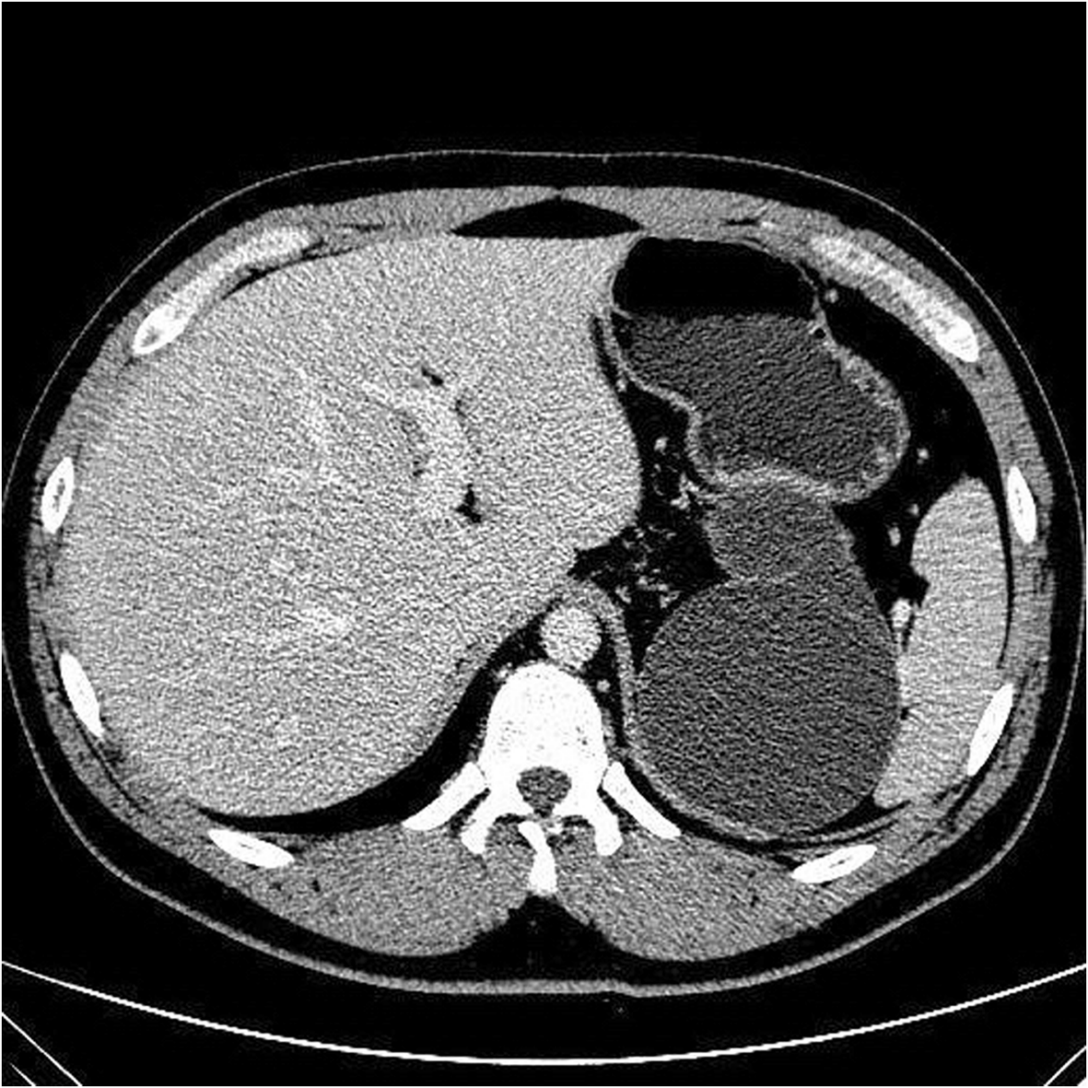
**Computed tomography of the abdomen (with contrast agent).** A well-defined oval lesion is seen immediately adjacent to the gastric corpus and left adrenal gland area. A linear septum can also be seen.

**Figure 2:**
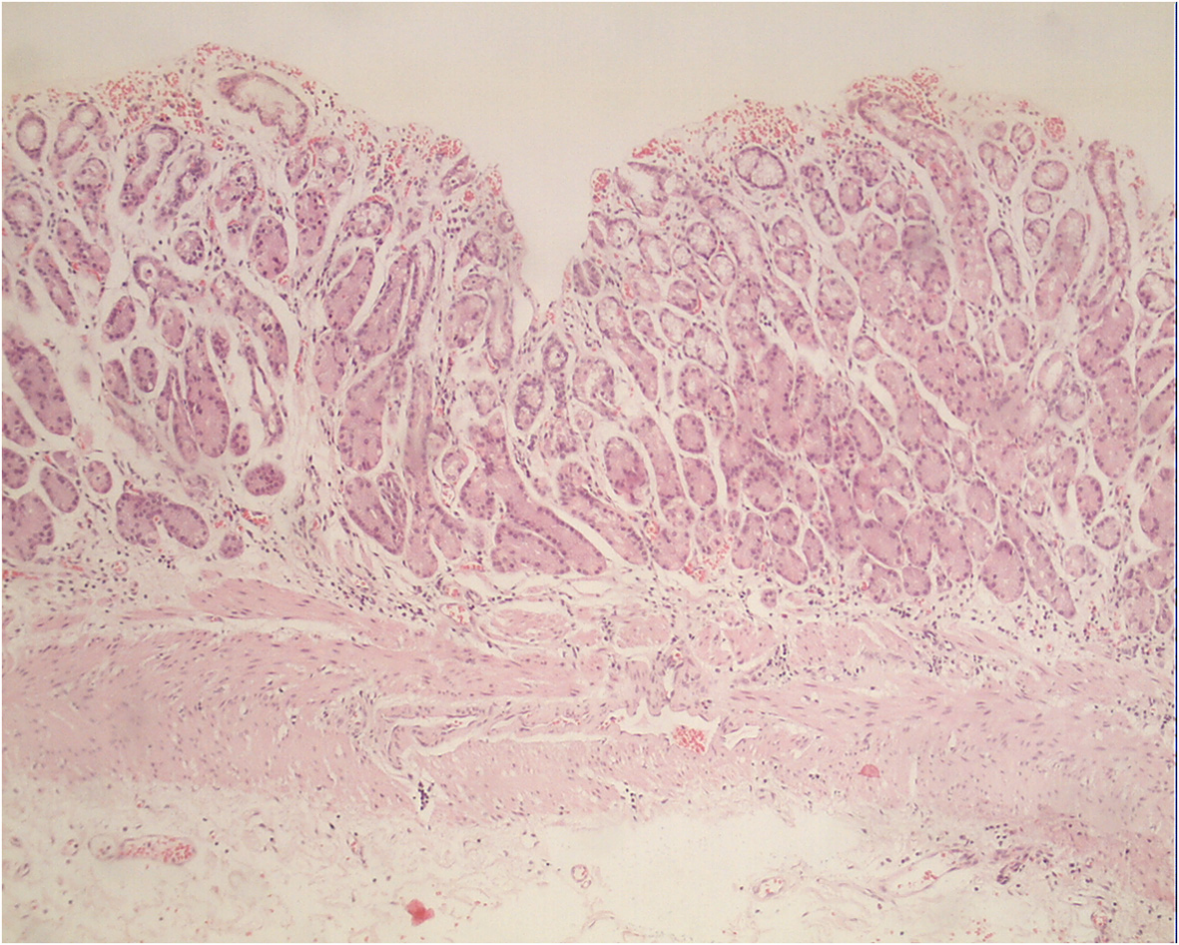
**Microscopic examination of the resected cystic lesion.** The gastric mucosa in the wall of the cystic lesion and a thick smooth muscular layer can be seen underneath (hematoxylin and eosin, ×100).

A 28-year-old man had been well until 8 months ago, when he visited his physician for a routine health screening. Abdominal sonography revealed a cystic lesion in close proximity to the left adrenal gland. Subsequent computed tomography (CT) of the abdomen confirmed the presence of the cystic lesion with no contrast enhancement (Figure 1). The cyst measured 8 × 13 × 12.5 cm and had a linear septum. Although the liver, gallbladder, spleen, and right adrenal gland were normal, the cyst seemed to cause a mass effect on the pancreas and the left kidney. However, no lymphadenopathy was noted. Laparoscopic retroperitoneal exploration was performed, and a 10 × 10 cm cystic lesion was found adhering to the gastric corpus. Unfortunately, attempts to remove the cystic lesion en bloc were not successful, and the lesion ruptured with the fluid contaminating the peritoneal cavity. The cystic lesion was removed grossly, and gross pathological examination showed a ruptured 7 × 6.5 × 4 cm irregular cyst with two cavities. Microscopic examination showed typical gastric mucosa with a smooth muscle component in the wall of the resected cyst with no malignancy (Figure 2). Therefore, a gastric duplication cyst was diagnosed based on the images and histology.

**Figure 3:**
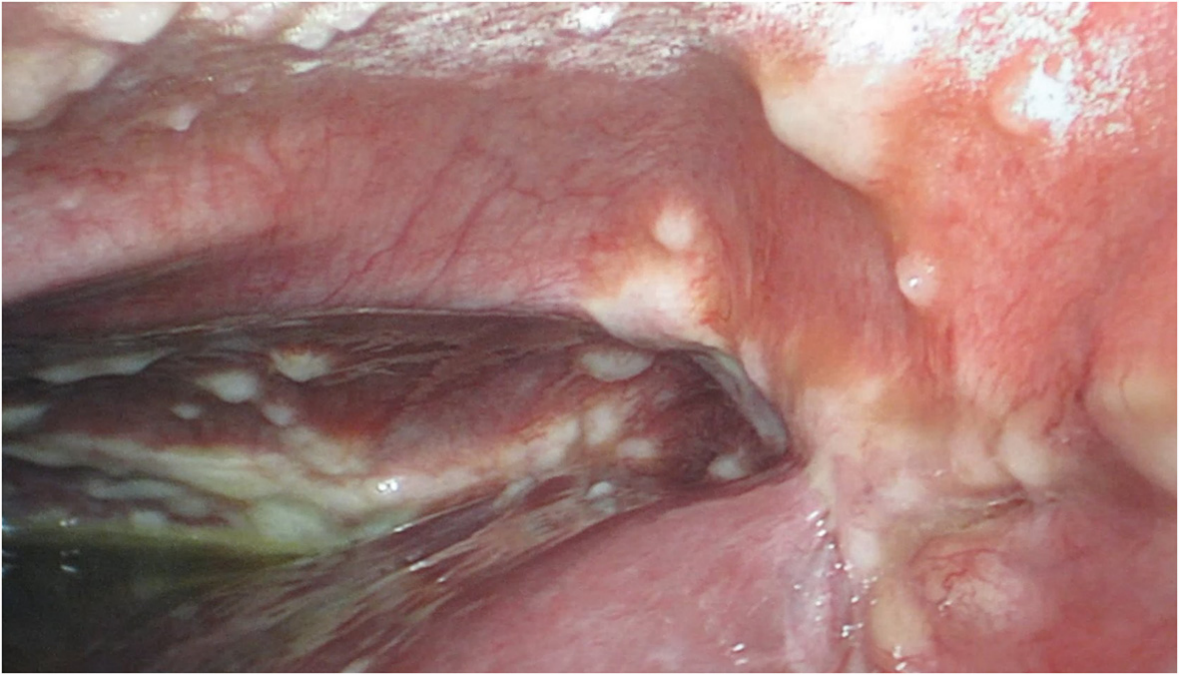
Diagnostic laparoscopic examination showing multiple white nodules on the surface of liver and peritoneum.

**Figure 4:**
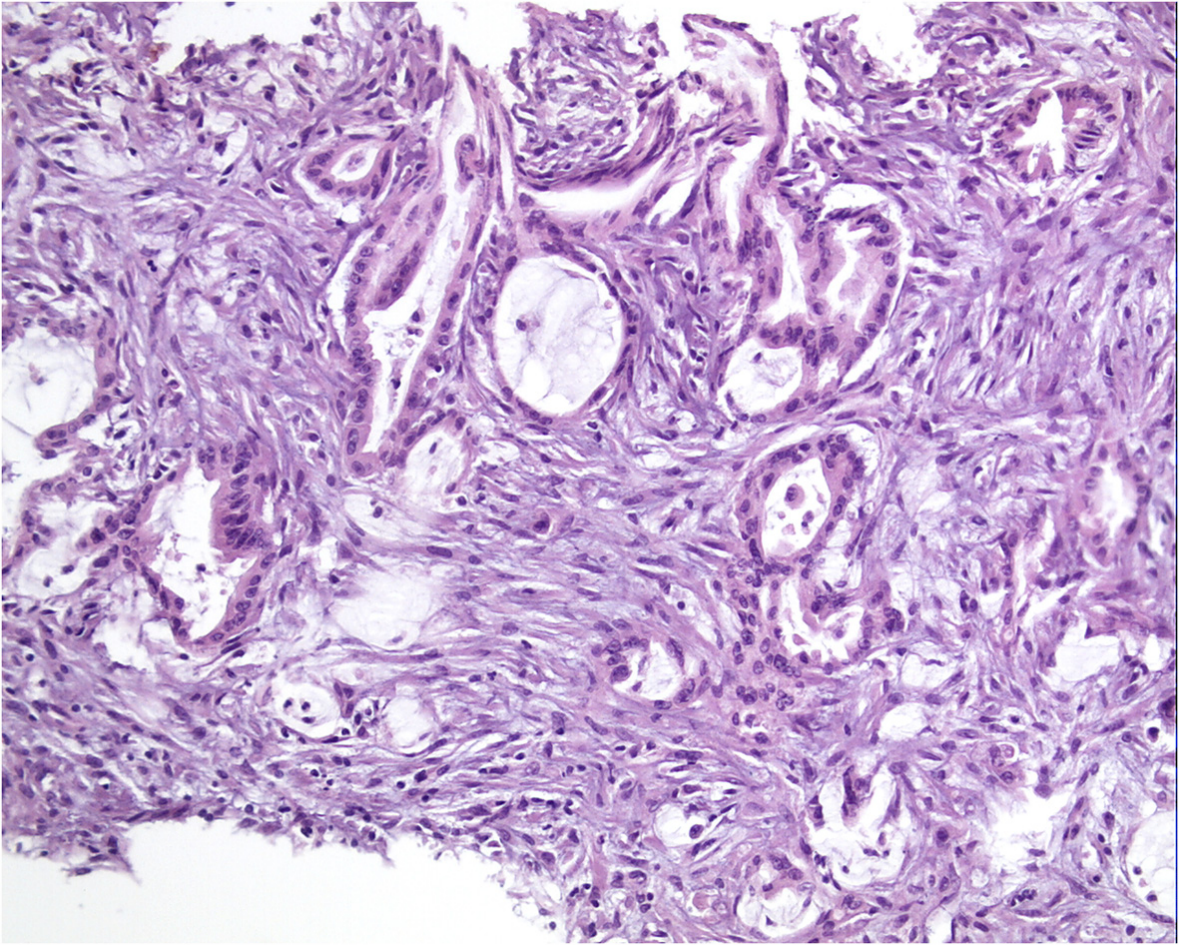
Histological features of the biopsied peritoneal nodules (hematoxylin and eosin, ×400).

Seven months after the surgery, the patient complained of abdominal distension as well as anorexia and dyspnea on exertion. A repeated abdominal CT scan showed a well-defined roundish low-intensity non-enhancing mass measuring 6.7 × 4.7 cm in the left adrenal area, along with massive ascites. On examination, the patient was found to be emaciated with positive shifting dullness. Laboratory examination showed normal liver function. Examination of serum tumor markers revealed that the carbohydrate antigen 19-9 (CA 19-9) level was 2145 U/L, while the levels of carbohydrate antigen 125 (CA 125) and carcinoembryonic antigen (CEA) were 209 U/L and 8.1 U/L, respectively. Peritoneal fluid analysis showed that it was yellow and turbid, and the exudates yielded a positive result in the Rivalta test. The levels of CA 19-9, CA 125, and CEA in the fluid were 12000 U/L, 1427.4 U/L, and 518 U/L, respectively. Acid-fast staining of the ascites yielded negative results. Multiple cytological analyses of the ascites revealed small nests of cells compatible with adenocarcinoma. Esophagogastroduodenoscopy, colonoscopy, and CT of the chest, abdomen, and pelvis failed to identify the primary lesion. A diagnostic laparoscopy showed multiple white nodules measuring 0.5- 1.5 cm scattered over the surface of liver, greater omentum, and peritoneum (Figure 3). Biopsy examination of the omental nodules indicated an adenocarcinoma infiltrating the adipose and fibrous tissues (Figure 4). Immunohistochemical staining yielded a positive result for markers of cytokeratin 20 (CK 20), CK 7 and P53, with a Ki-67 index of 40%, and AB-PAS (Alcian blue-periodic acid sthiff) staining was also positive. Thus, peritoneal carcinomatosis was diagnosed but no primary tumor was identified.

## Discussion

Gastrointestinal duplications are mostly diagnosed during the first year of life [2], although occasionally, gastric duplication cysts have been detected during health screening in adults, as was observed in the present study and that by Zheng et al. [2]. Kremer et al. [3] described 9 cases, of which only one involved an adult patient. In gastrointestinal duplications, the most commonly involved organs are the small intestine (45%) and esophagus (19%) [4]. Gastric duplications are relatively infrequent, accounting for about 9% of the reported cases [4]. Currently, it is thought that the pathogenesis of gastrointestinal duplications has a multivariate embryologic basis. Rowling et al. proposed the following diagnostic criteria for gastrointestinal duplications: (1) Grossly, they are spherical cysts or tubular structures located in, or immediately adjacent to, part of the gastrointestinal tract. (2) They are surrounded by at least one coat of smooth muscle, fusing with the muscularis propria of the alimentary canal (usually all three muscle layers are present, without a plane of cleavage between the cyst and the alimentary canal). (3) They are lined by typical gastrointestinal mucosa [4]. In our case, postoperative pathological examination confirmed the presence of the gastric mucosa and smooth muscle in the cyst wall (Figure 2), both of which are criteria for the diagnosis of gastric duplication cysts.

Gastric duplication cysts are typically found in the greater curvature of stomach and always manifest as non-communicating oval cysts [2]. The symptoms are usually not specific and include abdominal pain, vomiting, and weight loss [5]. Some patients experience acute onset of bleeding and pancreatitis [6-8]. In a study of 83 patients with gastric duplications, Pruksapong et al. [5] reported more than 50% cases with manifestations of a palpable abdominal mass (55/83) and vomiting (53/83). Vomiting is usually explained by partial or complete obstruction of the gastric outlet [9].

Preoperative diagnosis of gastric duplication cysts is always challenging. Differential diagnosis includes tumors of the stomach, especially gastric submucosal tumors [10], and cystic masses in adjacent organs, such as pseudocysts and mucinous cystic tumors of pancreas [11]. Due to the proximity of the stomach and adrenal gland, these lesions are often misdiagnosed as adrenal masses [12]. CT may assist in the evaluation of the cyst size and its anatomical relationship with the other organs [13]. However, histological examination remains the most important method for establishing the tissue origin of duplication cysts. In the present case, as shown on CT, the cystic lesion was in close proximity to both the stomach wall as well as the left adrenal gland. The intraoperative finding of the cyst adhering to the gastric wall and the characteristics of the gastric mucosa in the lumen of the cyst confirmed the diagnosis of gastric duplication cyst.

As agreed by most researchers, gastric duplication cysts have malignant potential, for which the underlying mechanism is still unknown. Since they have mostly been reported in children, the malignant transformation tends to be underestimated due to surgical removal of the cyst after diagnosis. In contrast, detection of this lesion in adults should raise greater alarm regarding malignancy. Survey of the literature has revealed only 9 cases of malignant transformation in adults reported to date [2,9,10,14-18]; these cases have been summarized in Table 1. As shown in the table, carcinomas arising from gastric duplication cysts generally occur in middle-aged adults, with a median age of 56 years and an age range of 25–71 years. The diameter of the duplication cyst ranges from 3.2 to 17.0 cm, with a median of 8 cm. The symptoms mainly include abdominal pain, weight loss, and vomiting, which are similar to those of benign cysts.

**Table 1:** Clinicopathological characteristics for cases of malignancy arising in gastric duplication cysts

**Reference**	**Sex/age (yr)**	**Symptoms**	**Cyst size (cm)**	**Macroscopic appearance**	**Pathology**	**Invasion**	**Follow up**
**Mayo et al.**[14]	F/64	Weakness, anorexia, weight loss	6	1.5 cm, Polypoid	Adenocarcinoma	Muscular layer of stomach	NED at 12 months
**Kuraoka et al.**[15]	M/56	Vomiting, weight loss	10	0.7 cm, Superficial slightly depressed	Well differentiated adenocarcinoma	Mucosa of the cyst	NED at 28 months
**Coit et al.**[16]	F/72	Abdominal pain, weight loss	4	Granular	Mucinous papillary adenocarcinoma	Submucosa of stomach	NED at 72 months
**Treiger et al.**[17]	M/50	Vomiting, weight loss	17	Ulcerative tumor	Infiltrating epithelial carcinoma	Unknown	Unknown
**Mamiya et al.**[18]	F/71	Abdominal pain, appetite loss	8	2.0 cm, Superficial slightly raised	Papillary adenocarcinoma	Wall of the cyst	NED at 1 month
**Kuraoka et al.**[15]	M/40	Fever, back pain	7	Multi focal, granular	Well differentiated tubular or papillary adenocarcinoma	Whole wall of the stomach	Liver metastasis at 7 months
**Horne et al.**[10]	M/40	Abdominal pain	12	5.5 cm nodule	Neuroendocrine carcinoma	Wall of the cyst	Multiple metastasis at 14 months
**Barussaud et al.**[9]	F/67	Abdominal mass, weight loss	NA	Unknown	Mixed adenocarcinoma and squamous cell carcinoma	Wall of the stomach and cyst	Peritoneal metastasis on presentation, liver metastasis at 6 month
**Zheng et al.**[2]	M/25	Asytomptomatic	8	3.0 cm nodule	adenocarcinoma	Wall of the stomach and cyst	NED/13

NED denotes no evidence of disease.

The present case study pertains to an asymptomatic patient who was incidentally diagnosed with a gastrointestinal duplication cyst by ultrasonography, similar to the patient described by Zheng et al. [2]. Only one case described by Barussaud et al. [11] has reported metastasis in the peritoneum in the initial diagnosis followed by liver metastasis 6 months later, even though the patient underwent radical distal gastrectomy. Two reports [10,15] documented metastasis during postoperative follow-up after surgery: one was of liver metastasis at 7 months, while the other was of multiple metastases at 14 months. Adenocarcinoma is probably the most common histologic type of gastric duplication cysts, since it has been observed in 6 of the 9 cases reported [2,14-16,18]. Other histologic studies revealed one case of neuroendocrine carcinoma [10], one case of both adenocarcinoma and squamous cell carcinoma [9], and one of epithelial carcinoma [17].

In our case, diagnosis of malignancy in the gastrointestinal duplication cyst was based on clinical suspicion rather than histologic confirmation, unlike the cases summarized above. Moreover, there were several findings to support our suspicion: firstly, The patient had been well until the duplication cyst was detected, with no signs of underlying malignancy; secondly, The cyst ruptured during initial surgery resulting in its incomplete removal, with the advent of peritoneal http://metastastic adenoma noted several months later; lastly, the subsequent overall evaluation failed to identify any primary tumor, and the only findings were remnant cystic lesions and peritoneal adenocarcinoma. As seen in the cases presented in Table 1, the malignancy of the gastric duplication cysts always manifests as relatively small nodules and slightly raised or depressed lesions, which may not be detected on a preoperative CT scan. Thus, it is likely that the duplication cysts may have already transformed into malignant cysts prior to the surgical attempt, and the part that was not removed contained the malignant tissue. The rupture of the cyst possibly contaminated the peritoneal cavity with the fluid containing malignant cells, resulting in peritoneal carcinomatosis.

In our opinion, the metastatic adenocarcinoma with peritoneal carcinomatosis in this young male patient raised an important question about appropriate management of patients with gastric duplication cysts. Accurate preoperative diagnosis and optimal surgical management are the crucial lessons from this case. Some researchers have recommended that once a duplication cyst has been diagnosed, even if it is an asymptomatic one, it should be removed and managed like a malignant tumor [2,9]. Our case validates this recommendation and additionally highlights the need for adequate surgical removal and postoperative histologic evaluation.

## Conclusions

In summary, we report a case of a gastric duplication cyst and peritoneal metastatic adenocarcinoma in a young adult man. The malignancy probably developed from the gastric duplication cyst, as indicated by the clinical presentation and chronological developments. We recommend that the possibility of malignant transformation be considered during surgical evaluation of adult patients with gastrointestinal duplication cysts.

## Consent

Written informed consent was obtained from the patient for publication of this Case report and any accompanying images. A copy of the written consent is available for review by the Editor of this journal.

## Competing interests

The authors declare that they have no competing interest.

## Authors’ contributions

LXC participated in the management of this case, designed this report , collected the materials of this manuscript and revised the manuscript; LKL participated in management of this case , collected the materials of this manuscript and drafted the manuscript; WJ and MMM participated in the management of this case and processing of materials in this manuscript; LH, SH and TWP participated in the management of this patient; CH contributed in the pathological diagnosis of this patient and supplied the pathological materials. All authors read and approved the final manuscript.

## Pre-publication history

The pre-publication history for this paper can be accessed here:

http://www.biomedcentral.com/1471-230X/14/48/prepub
